# A Positive Feedback Amplifier Circuit That Regulates Interferon (IFN)-Stimulated Gene Expression and Controls Type I and Type II IFN Responses

**DOI:** 10.3389/fimmu.2018.01135

**Published:** 2018-05-28

**Authors:** Agata Michalska, Katarzyna Blaszczyk, Joanna Wesoly, Hans A. R. Bluyssen

**Affiliations:** ^1^Department of Human Molecular Genetics, Faculty of Biology, Institute of Molecular Biology and Biotechnology, Adam Mickiewicz University, Poznan, Poland; ^2^Laboratory of High Throughput Technologies, Faculty of Biology, Institute of Molecular Biology and Biotechnology, Adam Mickiewicz University, Poznan, Poland

**Keywords:** interferon, JAK/signal transducer and activator of transcription signaling pathway, signal transducer and activator of transcriptions, interferon-stimulated gene factor 3, interferon regulatory factor 1, transcriptional regulation, antiviral activity

## Abstract

Interferon (IFN)-I and IFN-II both induce IFN-stimulated gene (ISG) expression through Janus kinase (JAK)-dependent phosphorylation of signal transducer and activator of transcription (STAT) 1 and STAT2. STAT1 homodimers, known as γ-activated factor (GAF), activate transcription in response to all types of IFNs by direct binding to IFN-II activation site (γ-activated sequence)-containing genes. Association of interferon regulatory factor (IRF) 9 with STAT1–STAT2 heterodimers [known as interferon-stimulated gene factor 3 (ISGF3)] or with STAT2 homodimers (STAT2/IRF9) in response to IFN-I, redirects these complexes to a distinct group of target genes harboring the interferon-stimulated response element (ISRE). Similarly, IRF1 regulates expression of ISGs in response to IFN-I and IFN-II by directly binding the ISRE or IRF-responsive element. In addition, evidence is accumulating for an IFN-independent and -dependent role of unphosphorylated STAT1 and STAT2, with or without IRF9, and IRF1 in basal as well as long-term ISG expression. This review provides insight into the existence of an intracellular amplifier circuit regulating ISG expression and controlling long-term cellular responsiveness to IFN-I and IFN-II. The exact timely steps that take place during IFN-activated feedback regulation and the control of ISG transcription and long-term cellular responsiveness to IFN-I and IFN-II is currently not clear. Based on existing literature and our novel data, we predict the existence of a multifaceted intracellular amplifier circuit that depends on unphosphorylated and phosphorylated ISGF3 and GAF complexes and IRF1. In a combinatorial and timely fashion, these complexes mediate prolonged ISG expression and control cellular responsiveness to IFN-I and IFN-II. This proposed intracellular amplifier circuit also provides a molecular explanation for the existing overlap between IFN-I and IFN-II activated ISG expression.

## Introduction

Interferons (IFNs) belong to the superfamily of cytokines that were discovered by Isaacs and Lindenmann as antiviral proteins (1). Since then it has become clear that IFNs do much more than inhibiting virus replication, and are also involved in cell proliferation, apoptosis, inflammation, as well as adaptive immunity (2–4).

Interferons comprise a family of molecules divided into three main sub families: IFN-I, IFN-II, and IFN-III. IFN-II is also known as IFNγ that binds the IFNγ receptor (IFNGR) complex and mediates broad immune responses to non-viral pathogens. IFN-II is mainly produced in response to foreign antigens or mitogens by T lymphocytes and natural killer (NK) cells. IFN-I predominantly consists of IFNα and IFNβ subtypes and can be produced by many substances in a variety of cell types. However, viruses and synthetic double-stranded RNAs are the most potent inducers of IFN-I. They engage the ubiquitously expressed IFNα receptor (IFNAR) complex and are known to be crucial for activating a robust host response against viral infection (2–4). IFN-III contains the subtypes IFNλ1, IFNλ2, IFNλ3 (5), and the recently discovered IFNλ4 (6). These IFNs signal through a receptor complex consisting of IL10R2 and IFNLR1 and possess potent antiviral activity (5).

Interferon-I and IFN-II both induce IFN-stimulated gene (ISG) expression through Janus kinase (JAK)-dependent phosphorylation of signal transducer and activator of transcription (STAT) 1 and STAT2. STAT1 homodimers, known as γ-activated factor (GAF), activate transcription in response to all types of IFNs by direct binding to IFN-II activation site [γ-activated sequence (GAS)]-containing genes. Association of interferon regulatory factor (IRF) 9 with STAT1–STAT2 heterodimers [known as interferon-stimulated gene factor 3 (ISGF3)] or with STAT2 homodimers (STAT2/IRF9) in response to IFN-I, redirects these complexes to a distinct group of target genes harboring the interferon-stimulated response element (ISRE) (7). Similarly, IRF1 can regulate expression of ISGs in response to IFN-I and IFN-II by directly binding the ISRE or IRF-responsive element (IRE) (8, 9). The partially overlapping and differential activation of transcription factor complexes and regulation of target gene expression by IFN-I and IFN-II, may be a consequence of the biological similarities and differences of these two types of IFN.

According to the general paradigm, phosphorylation of STAT1 and STAT2 in response to IFN-I and or IFN-II displays a robust and transient character. This is followed by a similar ISG expression pattern that decreases over time. However, recent studies have shown that IFN signaling is much more complex and revealed that ISG expression patterns are globally sustained in response to both types of IFN (10–12). This sustained response relies on prolonged expression of the ISGF3 and GAF components STAT1, STAT2 and IRF9 and IRF1 as part of a positive feedback loop. In addition, evidence is accumulating for a role of U-STAT1 and U-STAT2, with or without IRF9, and IRF1 in basal as well as long-term ISG expression.

This review combines our latest findings with recent literature to provide insight into the existence of an intracellular amplifier circuit regulating ISG expression and controlling cellular responsiveness to IFN-I and IFN-II. Especially, we focus on how this feedback system regulates ISG transcription from the basal to the IFN-induced state at the genome-wide level, how it depends on phosphorylation and expression of ISGF3, IRF1, and GAF components, and how it controls cellular responsiveness to IFN-I and IFN-II in relation to antiviral activity.

## Phosphorylated STAT1- and STAT2-Dependent ISG Transcription: ISGF3, STAT2/IRF9, and GAF

### ISGF3: Phosphorylated STAT1 and STAT2 with IRF9

All IFN-I subtypes bind the IFNAR1 and IFNAR2 subunits of the heterodimeric transmembrane IFNAR receptor to activate the JAK/STAT pathway, used by many cytokines and growth factors. The main constituents of this pathway are: (I) the JAK family of non-receptor tyrosine kinases JAK1, JAK2, JAK3, and tyrosine kinase 2 (TYK2) (13) and (II) transcription factors of the STAT family STAT1–STAT6. STATs are characterized by seven structurally and functionally conserved regions: the N-terminal domain, coiled-coil domain, DNA-binding domain, linker domain, Src-homology 2 domain, tyrosine phosphorylation site, and transcriptional activation domain (13–15).

**Figure 1:**
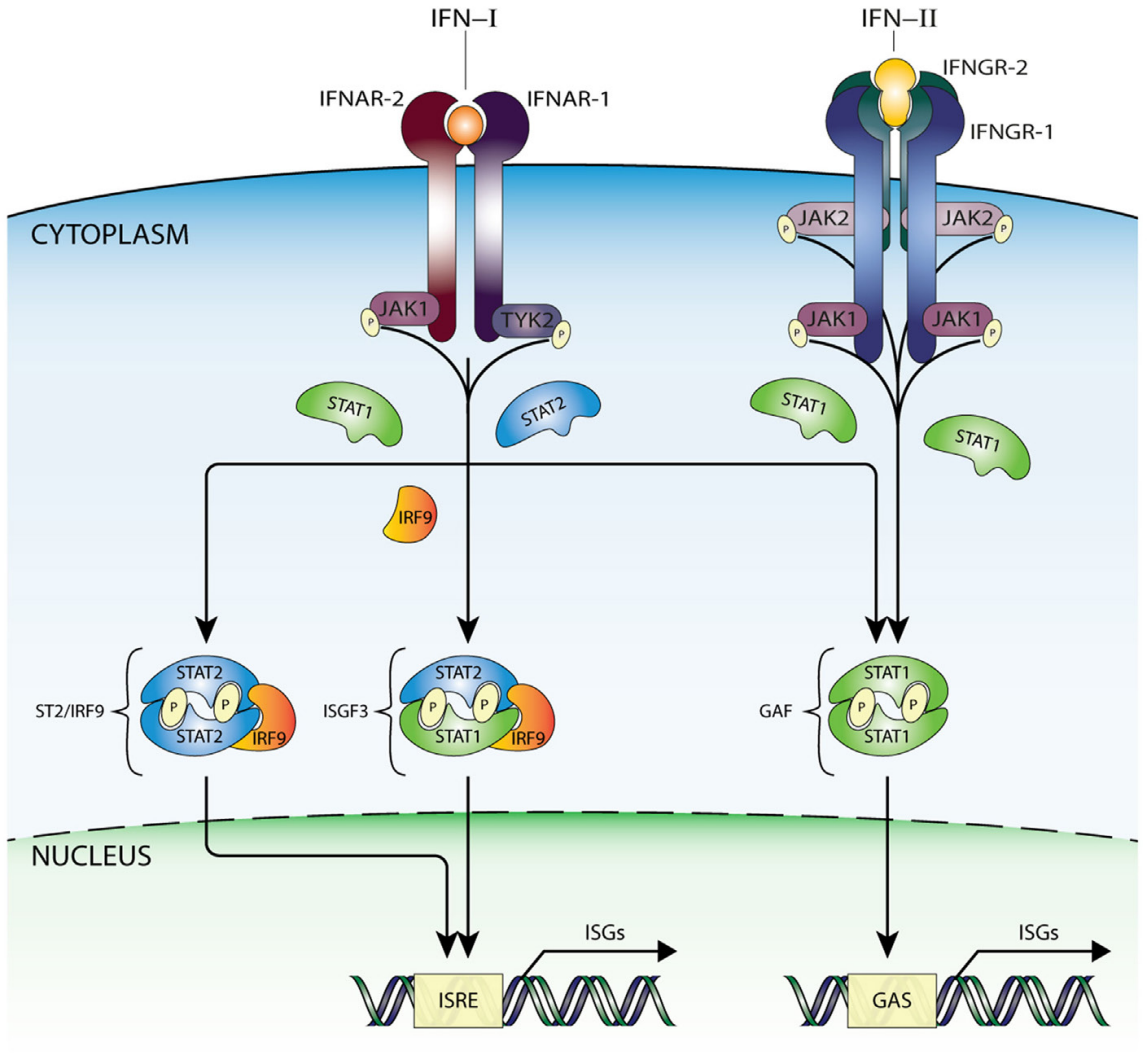
IFN-activated ISG transcription mediated by ISGF3, GAF, and STAT2/IRF9 complexes. IFN-I is recognized by a heterodimeric receptor composed of IFNAR1 and IFNAR2 subunits. After IFN binding and receptor dimerization, juxtapositioning of JAK1 and TYK2 results in increased kinase activity *via* transphosphorylation and subsequent STAT protein recruitment. Receptor-bound STAT proteins are successively phosphorylated, dimerize, and translocate to nucleus, where ISG transcription is initiated after binding ISRE or GAS sites. Thus, in response to IFN-I three active complexes are formed that play a crucial role in transcriptional regulation. A STAT1/STAT2 heterodimer associated with IRF9, known as ISGF3, binds the ISRE motif present in >300 ISGs. Second, with the same mode of action, an alternative complex built of STAT2 homodimers and IRF9 (STAT2/IRF9). In addition, STAT1 homodimers (known as GAF), which specifically recognize the GAS sequence. On the other hand, IFN-II interacts with a different receptor built of two IFNGR1 and two IFNGR2 subunits connected with JAK1 and JAK2 kinases, which are capable of phosphorylating only STAT1 proteins, resulting in dimerization and formation of GAF. GAF translocates to the nucleus and targets GAS-containing genes, in a similar way as in response to IFN-I. Abbreviations: IFN, interferon; STAT, signal transducer and activator of transcription; IRF, interferon regulatory factor; JAK, Janus kinase; TYK, tyrosine kinase; ISGF3, interferon-stimulated gene factor 3; GAF, γ-activated factor; ISRE, interferon-stimulated response element; GAS, γ-activated sequence; ISG, interferon-stimulated gene; P, phosphate; IFNAR, IFNα receptor.

Interferon-I binding to IFNAR results in receptor dimerization and increased JAK1 and TYK2 kinase activity *via* juxtapositioning and transphosphorylation (13). Subsequently, JAK1 and TYK2 phosphorylate IFNAR1 and IFNAR2 on target tyrosine residues that become docking sites for STAT1 and STAT2 (14). Receptor-bound STAT1 and STAT2 are thus phosphorylated on a critical tyrosine residue (pTyr) driving SH2-pTyr mediated dimer formation, nuclear translocation, and transcriptional activation. In the canonical pathway of IFN-I-mediated signaling, Tyr701 phosphorylation of STAT1 and Tyr690 of STAT2 leads to heterodimerization, interaction with IRF9 and formation of ISGF3 (Figure 1). After translocation to the nucleus, this complex binds the ISRE (consensus sequence AGTTTCN_2_TTTCN) of over 300 ISGs, such as *ISG15, OAS1-3, IFIT1-3*, or *MX1* and *2* that are instrumental in antiviral activity (13–15) (Figure 1).

The basic function of the ISGF3-dependent response is to mediate rapid and robust IFN-I responses by regulating transient transcription of antiviral ISGs (16). This fast and large-scale response enables to combat with infection, but simultaneously prevents long-term harmful effects to activated cells. For this reason, the ISGF3-dependent response is in general time-limited following a quick assembly of the complex from its pre-existing components and its transport to the nucleus where it binds to ISRE-containing ISGs. In this respect, STAT2 is constantly imported to the nucleus in an unphosphorylated state due to its association with IRF9 that contains a strong nuclear localization signal (NLS). The dominant nuclear export signal (NES) of STAT2 shuttles the complex back to the cytoplasm. Following STAT2 tyrosine phosphorylation, it can form dimers with STAT1 and the trimeric ISGF3 complex, and together with the NLS and NES present in STAT1 nucleocytoplasmic shuttling of ISGF3 and its components is controlled in a timely and spatial fashion (17). In 1989, Levy et al. provided evidence that the active ISGF3 complex is already detectable within 2 min after exposure of cells to IFNα (18). Thus, the rapid ISGF3 assembly serves fast and robust IFNα responses that are diminished in time and coincide with the phosphorylation profiles of STAT1 and STAT2 (19). In this process, important negative feedback mechanisms collaborate to dampen STAT phosphorylation and ISG expression several hours after IFN stimulation. These include members of the suppressors of cytokine signaling protein family (SOCS), in particular SOCS1. SOCS1 acts like a negative feedback loop for IFN-I signaling in a STAT1-dependent manner, by inhibiting JAK tyrosine kinase activity directly through its kinase inhibitory region (20).

### STAT2/IRF9: Phosphorylated STAT2 with IRF9

Previously, we revealed that in the absence of STAT1, STAT2 homodimers interact with IRF9 that form the ISGF3-like complex STAT2/IRF9 and activate transcription of ISRE-containing genes in response to IFNα (21) (Figure 1). Under similar conditions, it was shown that an IRF9–STAT2 hybrid protein reinstates interferon-stimulated gene expression (22, 23). Multiple studies have subsequently provided evidence for the existence of a STAT1-independent IFN-I signaling pathway, where STAT2/IRF9 substitutes ISGF3 function (24–27). STAT2 has been shown to heterodimerize with other STATs than STAT1. For example, in U266 cells STAT2 specifically interacted with STAT3 in an IFN-I-dependent manner in the presence of STAT1 (28). So far, however, no biochemical evidence exists that this complex together with IRF9 can reconstitute “ISGF3-like” functions in the absence of STAT1. Proof has been provided for an “ISGF3-like” role of STAT2–STAT6 heterodimers complexed with IRF9, but this complex seems to have a restricted role in B-cell specific IFN-I signaling (29).

Recently, more detailed insight was provided into the genome-wide transcriptional regulation and the biological implications of STAT2/IRF9-dependent IFNα signaling as compared with interferon-stimulated gene factor 3 (ISGF3). In STAT2 overexpressing STAT1-deficient human and mouse cells, IFNα-induced expression of typical ISGs correlated with the kinetics of STAT2 phosphorylation, and the presence of a STAT2/IRF9 complex. Results revealed that in the absence of STAT1, the STAT2/IRF9 complex triggered expression of a similar subset of ISGs as ISGF3 (30) which is consistent with the observations of Lou et al. (31). It is also in agreement with the ability of STAT2 and IRF9 to move in and out of the nucleus as a complex (17). Among these commonly upregulated ISGs were known genes involved in antiviral response and within the promoters of all of these genes, we confirmed the presence of a classical ISGF3-binding ISRE (Figure 1). Interestingly, these genes exhibited different expression profiles: early and transient when driven by ISGF3 and delayed and prolonged expression when driven by STAT2/IRF9. This also correlated with the transient ISRE binding pattern of ISGF3 components as compared to the more prolonged binding of STAT2/IRF9 (Blaszczyk et al., manuscript in preparation). In this respect, Abdul-Sater et al. (32) showed that a prolonged activity of JAK1 and reduced levels of SOCS1 associated with the delayed kinetics of STAT2 activation (33). In human and mouse STAT1 KO cells overexpressing STAT2 cells (30) (data not shown) as well as in STAT1 KO BMM cells (32), reduced SOCS1 expression enables STAT2/IRF9-dependent and delayed expression of a subset of antiviral ISGs.

Our experiments also offered additional proof for the functional overlap between STAT2/IRF9 and ISGF3, by showing that the STAT2/IRF9 complex was able to trigger an antiviral response upon encephalomyocarditis virus and vesicular stomatitis Indiana virus (30). Thus, STAT2/IRF9 exhibits a biological function in the reconstitution of the antiviral response in cells lacking STAT1. In line with this, Yamauchi et al. recently showed in Huh-7.5 cells that IFN-I responses were only partially attenuated by knockout of STAT1 but completely by knockout of STAT2. Moreover, they observed that IFN-I inhibited hepatitis C virus (HCV) replication in a STAT2-dependent but STAT1-independent manner (12).

Along the same lines, it was demonstrated that STAT2 plays a crucial role in a STAT1-independent protective mechanism against *L. pneumophila* and Denga virus infection. This highly suggests that DENV-mediated inactivation of STAT1 function alone is not sufficient to neutralize antiviral responses. More important, it is tempting to speculate that the STAT2/IRF9 pathway evolved as a backup response against pathogens that block STAT1 activity (32) [e.g., Paramyxovirus (34, 35) or Sendai virus (36)].

### GAF: Phosphorylated STAT1 Homodimers

It has become clear that IFN-I is also able to regulate expression of a distinct set of genes through an additional STAT-based signaling cascade that depends on the formation of STAT1 homodimers (13–15) (Figure 1). Accordingly, binding of IFN-II to its tetrameric receptor, composed of the subunits IFNGR1 (2×) and IFNGR2 (2×), first leads to transphosphorylation and activation of JAK1 and JAK2. These JAKs then phosphorylate specific tyrosine residues of the IFN-II receptor, which serve as STAT1 docking sites, but not STAT2. Thus, IFN-II specifically triggers the tyrosine phosphorylation of STAT1 and homodimer formation. Subsequently, STAT1 homodimers (known as GAF) translocate into the nucleus to activate genes containing the GAS DNA element (13–15) (Figure 1). The NLS and NES within the DNA-binding domain of STAT1 are essential both for its nucleocytoplasmic shuttling and for regulating the amount of transcriptionally active GAF in the nucleus (37). As mentioned above, STAT1 homodimers are also formed after IFN-I treatment (Figure 1), however, because of direct competition with STAT1–STAT2 heterodimerization and ISGF3 complex formation, in much lower amounts (38). Therefore, in most cell types, the IFN-I-dependent GAF-activated pathway is less potent as compared to that dependent on IFN-II, which does not activate STAT1–STAT2 heterodimer formation. The GAS element is a palindromic sequence [consensus: TTCN_(2–4)_GAA] that binds all STATs, except for STAT2. Thus, STAT1 homodimers bind to an element with canonical *N* = 3 spacing (39). To date, many GAS-containing STAT1-target genes have been identified (40), including guanylate-binding protein (*GBP*), *SOCS1, IRF1*, and *IRF8*.

Like STAT1 and STAT2 phosphorylation in response to IFN-I, STAT1 phosphorylation upon IFN-II stimulation displays a rapid raise that drops in time and correlates with a transient ISG expression profile. Among the upregulated proteins is SOCS1, which culminates in the reduction of STAT1 phosphorylation after IFN-II stimulation (20).

## Unphosphorylated STAT1 and STAT2: U-ISGF3, U-STAT2/IRF9, and U-STAT1

### IFN Dependent

**Figure 2:**
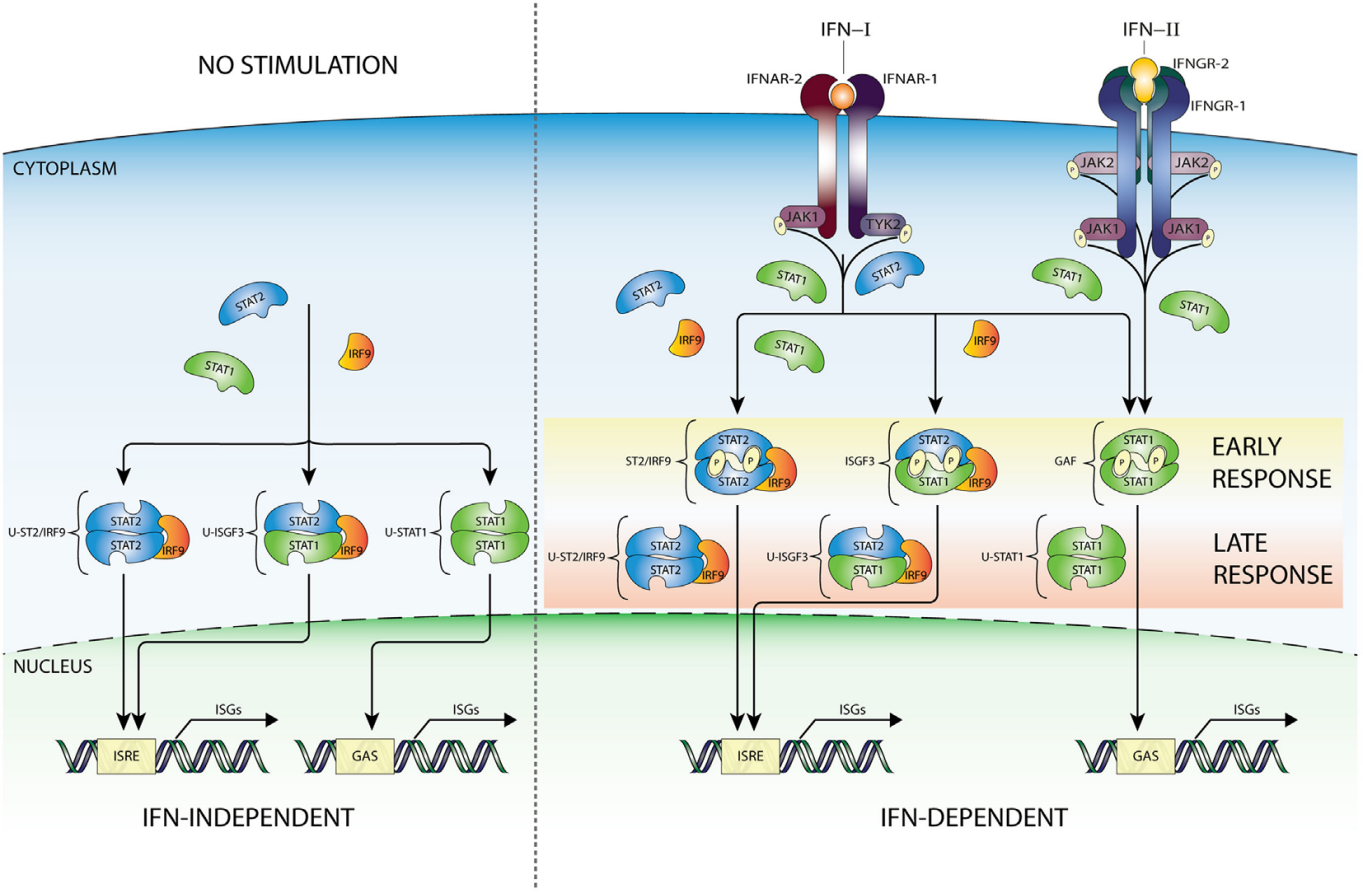
IFN-dependent and -independent ISG transcription mediated by complexes comprised unphosphorylated STATs. In addition to the classical IFN signaling pathway based on complexes of phosphorylated STAT proteins (early response—details Figure 1), evidence exists for the involvement of unphosphorylated versions of ISGF3 (U-ISGF3), GAF (U-STAT1), and STAT2/IRF9 (U-STAT2/IRF9), in maintaining basal expression of certain ISGs in unstimulated cells (IFN independent). In addition, these unphosphorylated complexes are instrumental in sustaining expression of ISGs in response to long-term IFN-I or IFN-II stimulation (IFN dependent), when the level of phosphorylated STAT proteins is decreasing, but *de novo* synthesized unphosphorylated STAT proteins accumulate and can be incorporated in unphosphorylated transcription factors (late response). Abbreviations: IFN, interferon; STAT, signal transducer and activator of transcription; IRF, interferon regulatory factor; JAK, Janus kinase; TYK, tyrosine kinase; ISGF3, interferon-stimulated gene factor 3; GAF, γ-activated factor; ISRE, interferon-stimulated response element; GAS, γ-activated sequence; ISG, interferon-stimulated gene; P, phosphate; U, unphosphorylated form.

Recent studies promote the model that U-STAT1 regulates expression of a subset of IFN-I-stimulated genes, including *IFI27, BST2, OAS1, OAS2, OAS3*, and *STAT1* itself. Many of the encoded proteins display antiviral and immune regulatory activities (2, 3, 10, 41–51). Supposedly, this occurred through the accumulation of newly synthesized STAT1 as part of a positive feedback loop after IFN stimulation (Figure 2) together with constantly replenished, but no longer phosphorylated, STAT proteins during process of nucleocytoplasmic shuttling (52). Moreover, prolonged exposure of cells to IFN-β was proposed to induce the expression of unphosphorylated STAT2 (U-STAT2) and IRF9 which together with U-STAT1 form unphosporylated ISGF3 (U-ISGF3) (19) (Figure 2). Upon nuclear translocation, U-ISGF3 subsequently maintained the expression of a subset of the initially induced ISRE-containing ISGs, resulting in prolonged resistance to virus infection and DNA damage. Interestingly, the U-ISGF3-dependent elongated expression of antiviral genes is possibly mediated by distinct ISREs (10).

A similar scenario was recently proposed for U-STAT2/IRF9 by Lou et al., in which abundance of U-STAT2 and IRF9 proteins mediated prolonged transcription of the *RIG-G* gene independent of STAT2 phosphorylation, through the autocrine/paracrine action of secreted IFNα activated in all-trans retinoic acid-treated cells (Figure 2). This is in agreement with the ability of U-STAT2/IRF9 to shuttle in and out of the nucleus (17). So far, it is not clear if in the absence of STAT1 this complex effects IFN-I-dependent expression of other ISRE-containing genes.

It was claimed that U-STAT1 does not activate gene expression as a homodimer (10, 53). However, Yao et al. recently observed in *Mycobacterium tuberculosis* (Mtb)-infected macrophages a rapid increase in phosphorylated STAT1, which quickly declined over a period of hours, but a continued increase of unphosphorylated U-STAT1 that persisted for several days. As such, U-STAT1 affected the expression of several immune-associated genes, and lowered sensitivity of macrophages to CD95-mediated apoptosis during *Mtb* infection (54). Likewise, Majoros et al. showed the importance of U-STAT1 in IFN-I-induced gene expression regulation and biological activity in mice expressing a Stat1Y701F mutant (19).

Therefore, in spite of virus-induced decline in IFN production, host cells can continue synthesizing antiviral proteins in a U-ISGF3-dependent manner, and possibly U-STAT2/IRF9 and U-STAT1, since even low concentrations of IFN-I can lead to increased expression of STAT1, STAT2, and IRF9 proteins (Figure 2). Thus, host cells can maintain at least some antiviral functions even after IFN synthesis decreases and phosphorylated signaling molecules are inactivated, by a supposedly tyrosine phosphorylation-independent mechanism. The expression of ISGF3 components is also highly increased in long-term IFN-II-treated cells (11), providing the possibility for the formation of U-ISGF3 and U-GAF as well. Consequently, it is tempting to speculate that a similar U-ISGF3- or U-STAT1-dependent mechanism exists involved in long-term IFN-II-treated cells (Figure 2).

### IFN Independent

Constitutive ISG expression in the absence of IFNs is known to be critical for cellular susceptibility to viral infection (2). In this respect, it has become clear that U-ISGF3, U-STAT1, and U-STAT2/IRF9 can also mediate constitutive IFN-independent expression of ISGs to protect against viral infection (Figure 2). It has been reported, in different cell types, that U-STAT1 (52) and U-STAT2 (17) in combination with IRF9 are in constant motion between the cytoplasm and nucleus (37) and in the form of U-ISGF3 can be responsible for the basal activity of ISG promoters. For example, Wang et al. showed that in cell lines, three-dimensional (3D) cultured primary intestinal and liver organoids, and liver tissues under homeostatic conditions, endogenous STAT1, STAT2, and IRF9 could be observed in the nucleus. Indeed, under conditions without detectable IFNs, constitutive ISG expression was mediated by U-ISGF3 and correlated with genome-wide U-STAT1 binding to selective ISG promoters. This process effectively conferred resistance to HCV and HEV infections to host cells and was independent of IFN production and the upstream elements of IFN signaling (55). In conclusion, in host cells under homeostatic conditions U-ISGF3 was able to sustain constitutive ISG transcription and antiviral immunity. In STAT1 KO cells overexpressing STAT2 and IRF9, we observed that U-STAT2/IRF9 increases basal expression of several ISGs including *IFI27, OAS2, OASL*, and *IFI44*. As mentioned above, in untreated cells, U-STAT2/IRF9 is able to shuttle between the cytoplasm and the nucleus, with the potential to bind DNA and regulate expression of a selection of ISGs. Therefore, we propose a comparable set-up in the absence of STAT1, that abundant U-STAT2 and IRF9 proteins can form a complex and drive ISG expression independent of IFN treatment [(30); Nowicka et al. data not shown].

Finally, basal DNA-binding of U-STAT1 is connected to its nuclear localization as well as the constitutive expression of some targets (56, 57). For example, U-STAT1 complexes with IRF1 at the *LMP2* and *TAP2* promoters and maintains its constitutive expression (56).

Thus, these observations suggest that both IFN-dependent and IFN-independent antiviral mechanisms are present simultaneously and act in a cooperative fashion (Figures 1 and 2).

## IRF1 in ISG Transcriptional Regulation

**Figure 3:**
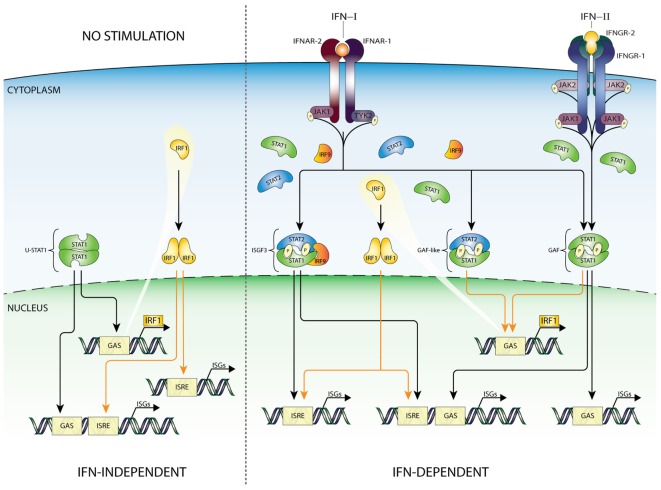
IFN-dependent and -independent ISG transcription mediated by IRF1. According to the current state of knowledge, expression of IRF1 is driven by homodimers of STAT1 (GAF), as well as STAT1/STAT2 heterodimers, recognizing a single GAS sequence in the IRF1 promoter. Recent studies confirmed that basal expression of IRF1 can be detected in many cell types under basal conditions. Moreover, binding of IRF1 to ISRE-containing genes can play a role in maintaining constitutive expression of certain group of ISGs (left panel). After IFN-I or IFN-II stimulation GAF and GAF-like (STAT1/STAT2 heterodimers) complexes are rapidly formed and translocated to the nucleus to initiate IRF1 expression. Subsequently, IRF1 as an additional abundant transcription factor can collaborate with ISGF3 and GAF complexes stimulating ISRE-containing genes, thus appearing as an important link between IFN-I and IFN-II responses. Abbreviations: IFN, interferon; STAT, signal transducer and activator of transcription; IRF, interferon regulatory factor; JAK, Janus kinase; TYK, tyrosine kinase; ISGF3, interferon-stimulated gene factor 3; GAF, γ-activated factor; ISRE, interferon-stimulated response element; GAS, γ-activated sequence; ISG, interferon-stimulated gene; P, phosphate.

Interferon regulatory factors are a family of 9 transcription factors (IRF1–9) which display a diversity in functions, including immune cell development cell cycle regulation, apoptosis, host defense, and oncogenesis (58, 59). IRFs all contain a conserved DNA-binding domain and IRF association domain. The DNA-binding domain consists of a five-tryptophan repeat that is located at the amino terminus and binds to a specific GAAA motif (IRF element: IRE), present within the promoters of IFNα and IFNβ genes as well as in ISGs (ISRE core). The IRE is a shorter version of the ISRE, not recognized by ISGF3. IRF1 and IRF9 are induced by both IFN-I and -II and viral infection (24, 58, 59). Like IRF9, IRF1 has also been shown to regulate transcription of ISGs in response to different types of IFNs. Nuclear localization of the IRF1 protein is regulated by an active NLS located immediately C-terminal to the DNA-binding domain (60). IFN-I and IFN-II induced transcriptional regulation of the IRF1 gene identified a single GAS element (TTTCCCCGAAA) and no ISRE in its proximal promoter (40), shown to bind GAF (Figure 3). As mentioned above, STAT1 homodimers are also formed after IFN-I treatment, however, usually in much lower amounts. Therefore, in most cell types IRF1 is more potently induced by IFN-II as compared to IFN-I (38). In mouse IRF9 KO embryonic fibroblasts (61) as well as in human IRF9 deficient U2A cells (62), IFN-I and IFN-II induction of IRF1 is not significantly different from Wt cells. On the other hand, IFN-I upregulation of IRF1 in U3C-STAT2 cells is severely hampered, consistent with the notion that GAF is a critical mediator and not ISGF3 or STAT2/IRF9 (10, 40, 63). Interestingly, in U2A cells, IFN-I-induced phoshoSTAT1–STAT2 heterodimers (pSTAT1–STAT2) were also shown to bind the *IRF1* GAS element and to act as a potent transcriptional activator of the *IRF1* gene (28, 62, 64–67) (Figure 3).

As described above, IRF9 complexes with STATs and redirects them to ISRE-containing ISGs. By contrast, IRF1 regulates ISG transcription through homo- or heterodimerization and binding to IRE or ISRE sequences. Thus, as a STAT1-target gene, IRF1 participates in secondary IFN-I and -II responses by activating transcription of ISRE-containing genes (Figure 3). Microarray analysis of IRF1 overexpressing Huh-7 and STAT1−/− fibroblasts identified many IRF1-target genes, including known antiviral ISGs as well as *STAT1, STAT2*, and *IRF9* (48). In a different study, it became clear that IRF1 restricts HEV replication by directly regulating expression of STAT1, resulting in increased levels of total and phosphorylated STAT1 protein and subsequently a panel of downstream antiviral STAT1-target genes (68). Furthermore, the antiviral activity of IRF1 was dependent on the JAK–STAT signaling pathway, but independent of IFN production. Together, this points to a functional overlap between IRF1 and ISGF3 and a role of IRF1 in regulating expression of ISGF3 components.

By generating mice deficient for both IRF9 and IRF1 alleles, Kimura et al. studied the functional interaction of IRF9 and IRF1 in IFN-I and IFN-II-induced gene regulation. For example, it was shown that IFN induction of the ISRE-containing genes *OAS* and *PKR* is primarily regulated by IRF9, in the context of ISGF3; i.e., IRF1 cannot compensate for the loss of IRF9 (61). Interestingly, induction of *GBP* by IFN-I required both IRF1 and IRF9, consistent inducible genes (69, 70). In contrast to IFN-I, IFN-II induction of *GBP* mRNA was largely dependent on IRF1. Thus, the *GBP* promoter differentially utilizes IRF9 and IRF1, depending on the type of IFN used to stimulate the cells.

Collectively, this highly suggested that IRF1 and ISGF3 are not functionally redundant but complement each other and collaborate to ensure the induction of the full range of overlapping target genes which respond to IFN-I and IFN-II (Figure 3).

Collaborations between IRF1 and STAT1 in transcriptional regulation of ISRE and GAS-containing ISGs have also been described (Figure 3). For example, the IFNγ-induced expression of *CIITA, GBP1*, and *gp19* was shown to depend on both STAT1 and IRF1 (71–73). Moreover, an independent study of 128 transcription factors in IFNγ treated K562 cells unraveled that STAT1–IRF1 co-binding is a general phenomenon (74). It is tempting to speculate that a similar mechanism of STAT1–IRF1 co-binding plays a role in IFN-I-mediated transcriptional responses (Figure 3).

In an IFN-independent manner, basal expression of IRF1 can be detected in many cell types. Correspondingly, constitutive binding of IRF1 was shown to regulate constitutive ISG expression, either alone or in combination with STAT1 (56, 75) (Figure 3).

## Germline Mutations of STAT1 and STAT2 and the Preservation of IFN Responses

### STAT1

In 2001, Dupuis et al. described three patients carrying a heterozygous germline L706S STAT1 mutation, which lead to an impaired ability to phosphorylate and translocate mutated STAT1 protein to the nucleus in response to IFN stimulation. Translocation of STAT2 according to immunofluorescence was not changed and expression of the ISFG3 target gene—*MXA* upon IFNα treatment was not reduced. These patients were susceptible to mycobacterial but not viral infections (76). Similar results were shown for patients with an autosomal dominant M654K STAT1 mutation with disrupted IFN-II response, but mildly influenced IFN-I response (77). Likewise, three other patients with identified STAT1 alleles (E320Q, Q463H, and L706S) that profoundly diminished IFN-II mediate ISG expression and responses to mycobacteria did not affect IFN-I-activated transcription and antiviral responses. Thus, indirect evidence exists that in patients with diminished STAT1 levels and/or function cellular mechanisms are present that allow preservation of intact antiviral responses (78).

On the other hand, a report about two unrelated infants with homozygous STAT1 deleterious mutations revealed for the first time that complete lack of STAT1 production abolishes not only IFNγ signaling but also IFNα/β response *in vivo*, which resulted in susceptibility and death after viral infection (79). This observation correlated with studies conducted on mouse STAT1−/− fibroblasts transfected with mutated L706S STAT1 alleles, showing disabled GAF and ISGF3 formation and disrupted translocation of the STAT2 protein to the nucleus in response to IFN. This was explained by possible sequestration of ISGF3 components by mutated STAT1 protein and its retention in the cytoplasm (76). It is also in agreement with later studies describing the phenomenon of mice with a homozygous mutation Y701F STAT1, which were more susceptible to infection than STAT1-null mice and were not able to develop a similar type of STAT2/IRF9-mediated response to IFN as seen in STAT1-null mice (19).

Apparently, not only loss-of-function STAT1 mutations are responsible for changed immunological responses. Unbalanced STAT1 hyperactivity in case of gain-of-function mutations also leads to the development of chronic mucocutaneous candidiasis or autoimmunological diseases (80).

### STAT2

Reported cases of STAT2 deficiency are always connected with a background of viral infections. Patients carrying STAT2 mutations are in general not able to induce full ISGF3-dependent IFN responses. The GAF-dependent IFN-I and IFN-II responses remain mostly unaffected. Moens et al. described a patient with a heterozygous STAT2 mutation who suffered from severe viral infections since infancy. Patient’s fibroblasts do not express STAT2 in full length or truncated form and after stimulation with IFNα ISRE-containing genes (*MX1, ISG15*, and *OAS1* were checked) were not induced. After transfection with wtSTAT2, cells restored IFN-inducible STAT2 phosphorylation and ISG expression (81). In another case study of siblings with STAT2 deficiency, the authors used whole-genome transcriptional profiling of patient’s fibroblasts with or without stimulation of IFNα. Only ~10% of upregulated genes in control cells were also upregulated in patient’s fibroblast. The residual upregulated ISGs were largely overlapping with these from control but expressed at lower level, except IRF1. Interestingly, promoter transcription factor binding site prediction revealed enrichment of GAF and/or IRF1 binding elements in the promoters of expressed ISGs (82). These observations suggest that type I IFN signaling (through ISGF3) is not completely essential for host defense against viral infections.

Together, from STAT1 and STAT2 germ-line mutation studies, it can be concluded that well-balanced and strictly controlled IFN-I and/or IFN-II-mediated immune responses are necessary but not sufficient to fight with infection. Moreover, potential backup systems, including STAT2/IRF9 or IRF1, could be activated under conditions where STAT1 or STAT2 are not fully active.

## Genome-Wide Binding of STAT1, STAT2, IRF9, and IRF1 and Regulation of ISRE and Gas-Dependent Transcription

Most of the knowledge about the DNA responsive elements involved in IFN-I and IFN-II signaling dates from early experiments that focused on individual genes and their role in the antiviral response (83). Accordingly, the ISRE was shown to exist in proximal ISG promoters as a single element or in multiple copies, in either orientation with (minor) consensus sequence variations (AGTTTCN2TTTCN; Table 1). Functional analysis of a selection of IFN-I-inducible genes (84, 85), as exemplified in Table 1, has revealed that ISRE is essential for IFN induction.

**Table 1:** ISRE and GAS sequences of known ISGs.

Type TFBS	Gene name	ISRE consensus sequence AG-TTT-CNN-TTT-CN	Linker^a^	GAS consensus sequence TTC-CNG-GAA	Location in relation to TSS	Reference
ISRE	MX1	AG-TTT-CGG-TTT-CA		–	Proximal	(86)
		GG-TTT-CG-TTT-CT		–	Intragenic	
		AG-TTT-CA-TTT-CT		–		

	ISG15	AG-TTT-CGG-TTT-CC		–	Proximal	(87)
		GG-TTT-CCC-TTT-CC		–		

	IFIT1	AG-TTT-CAC-TTT-CC		–	Proximal	(85)

	IFIT2	AG-TTT-CAC-TTT-CC		–	Proximal	(85)

	IFIT3	GG-TTT-CAT-TTT-CC		–	Proximal	(87)
		AG-TTT-CAC-TTT-CC		–		
		TG-TTT-CAG-TTT-CC		–	Intragenic	
		AG-TTT-CAC-TTT-CC		–		

	USP18	AG-TTT-CGC-TTT-CC		–	Proximal	(87)
		GC-TTT-CGT-TTT-CC		–		

	OAS1	GG-TTT-CG-TTT-CC		–	Proximal	(88)

	OAS2	AG-TTT-CAG-TTT-CC		–	Proximal	(89)

	OAS3	GG-TTT-CGT-TTT-CC		–	Proximal	(90)
		GC-TTT-CAG-TTT-CG		–		

	OASL	AG-TTT-CGA-TT-CT		–	Proximal	(91)

	ISG20	TG-TTT-CAG-TTT-CT			Proximal	(92)

GAS	IRF8	–		TTTC-TCG-GAAA	Proximal	(88)
		–		TTC-GAA-GAA		

	SOCS3	–		TTC-CTG-GAA	Proximal	(93)

	ICAM1	–		TTTC-CGG-GAAA	Proximal	(94)

	IRF1	–		TTTC-CCC-GAAA	Proximal	(95, 96)
		–		TTTC-TTA-TAAA	Distal −6 kb	^b^
		–		-TTC-CTG-GAAA		

ISRE/GAS	STAT1	AG-TTT-CGC-TTT-CC			Proximal	(97)
		CT-TTT-CGG-TTT-CC	32 nt	TTTC-CCC-GAAA	Distal −5.5 kb	

	STAT2	AG-TTT-CGG-TT-CC	15 nt	TTTT-CTC-GAA	Proximal	(98)
		CA-TTT-CTC-TTT-AT			Intronic	

	IRF9	AG-TTT-CAG-TT-CT	16 nt	TTTC-CCA-GAAA	Proximal	(87)

	IFITM1	AG-TTT-CTA-TTT-CC	16 nt	TTTC-TCA-GAA	Proximal	(87, 99)

	BST2	AG-TTT-CAG-TTT-CC	Overlap	TTTC-CCA-GAAA	Proximal	(100)

	TAP1	GA-TTT-CGC-TTT-CC	Overlap	TTTC-CCC-TAAA	Proximal	(101)

	SOCS1	GG-TTT-CAC-TTT-CA	1 nt	TTTC-CAA-GAAA	Distal −55 kb	(75)
		AC-TTT-CAG-TTT-CT	Overlap			

	IFI35	AC-TTT-CA-TTT-CC	Overlap	TTTC-CGT-GAAA	Proximal	(102)

	HLA-G	AG-TTT-CAC-TTT-CC	Overlap	TTTC-GA-GAA	Proximal	(103)

	ZC3HAV1	GC-TTT-TAG-TTT-CT	95 nt	-TTC-CCG-GAAA	Proximal	(91)

	AIM2^b^	AC-TTT-CGC-TT-GG	149 nt	TTTC-TGG-GAAA	Proximal	

	TRIM69^b^	GG-TTT-CTC-TTT-CT	14 nt	TTTC-CGA-GAAA	Distal −7.5 kb	

*^a^Distance between elements if both are present*.

*^b^Unpublished data*.

*ISG, interferon-stimulated gene; GAS, γ-activated sequence*.

Studies of the *GBP* gene, which is inducible by both IFNγ and IFNα/β, has led to the identification of the IFNγ activation site (GAS, consensus sequence: TTCCNGGAA (40); Table 1), in addition to an ISRE (104, 105). Subsequently, GAS was shown to be involved in the IFN-II-mediated transcriptional activation of several other genes and predominantly localized in their proximal promoters (Table 1). In the GBP gene, an ISRE was also found juxtaposed to the GAS, and both elements clearly contributed to both IFN-I and IFN-II responses (105, 106). Additional genes, stimulating cooperation of ISGF3 and GAF on ISRE and GAS composites have since been identified (40, 107) (Table 1). Cooperation between STAT1 and IRF1 in the IFNγ-regulation of GAS and ISRE-containing ISGs, has also been suggested (73, 107). In addition, binding modes exist in which multiple GAF complexes can be recruited to adjacent GAS sites (13, 108). Similar cooperative DNA-binding has been described for ISGF3 (109).

### IFN Dependent

More recent genome-wide binding approaches (using ChIP-chip or ChIP-seq), monitoring binding of STAT1, STAT2, and IRF1 in response to IFN-I or IFN-II, confirmed this genomic organization and identified many known and novel target genes with varying binding modes to individual or combined ISRE and GAS sites (83). For example, Hartman et al. used chromatin immunoprecipitation and DNA microarray analysis to identify STAT1 and STAT2-binding regions on chromosome 22 in IFN-treated cells (110). Markedly, non-conserved STAT1 occupancy was detected at GAS sites as well as novel STAT1 binding sites upon IFNα induction, not observed in IFNγ-treated cells. As expected, a large number of these sites correlated with STAT2 binding. However, others appeared STAT2 independent. Moreover, novel STAT2-binding sites could be identified, without STAT1, suggesting that under different activation conditions various mechanisms direct STAT1 and STAT2 binding to their targets.

**Figure 4:**
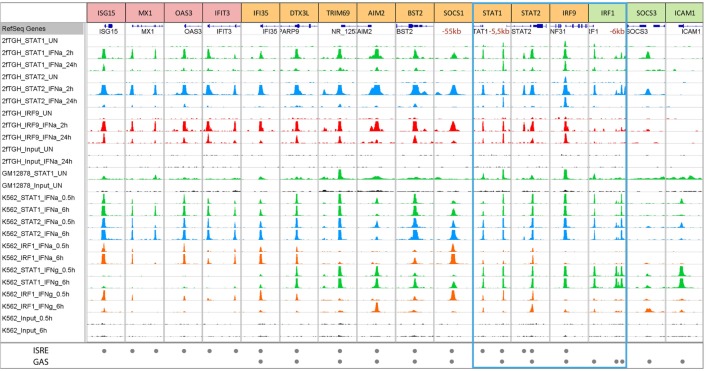
Representative modes of STAT1, STAT2, IRF9, and IRF1 binding to ISRE and GAS-containing ISGs in GM12878, K562, and 2fTGH cells treated with or without IFN-I or IFN-II. To further study genome-wide binding of STAT1, STAT2, IRF9, and IRF1, a ChIP-seq dataset from K562 cells treated with IFNα or IFNγ for 0.5 or 6 h and GM12878 cells used as a control were retrieved from the ENCODE database (GSE31477) and compared to our recently dataset from 2fTGH cells treated with or without IFNα for 2 and 24 h (unpublished data). Antibodies used in given experiments are indicated in the left panel and by color of the track—STAT1—green, STAT2—blue, IRF9—red, and IRF1—orange. Tracks derived from input sequencing are indicated in black. The bottom panel indicates ISRE or/and GAS sequences located under the peaks (see also Table 1). According to DNA motifs, ISGs can be divided into three groups: only ISRE-containing genes (examples are presented in red boxes—ISG15, MX1, OAS3, and IFIT3), only GAS-containing genes (examples in green boxes—IRF1, SOCS3, and ICAM1), and genes with both sequences (examples in yellow boxes—IFI35, DTX3L, TRIM69, AIM2, BST2, SOCS1, STAT1, STAT2, and IRF9). The blue frame marks components of ISGF3, GAF, and IRF1. Each group of genes displays differences in binding after IFN stimulation. ISRE-only genes show strong binding of components of ISGF3 (STAT1, STAT2, and IRF9) after IFNα, as well as IRF1 after IFNα and IFNγ, but no binding of STAT1 after IFNγ treatment. On the other hand, GAS-only genes demonstrate strong binding of STAT1 and STAT2 (components of GAF and GAF-like complexes) after IFNγ and IFNα treatment. The third group consists of ISRE + GAS composite-site containing ISGs, which display binding of all the components in response to both types of IFNs pointing to the possible cooperation and mechanism of co-binding of ISGF3, GAF and IRF1 for optimal gene expression. Abbreviations: IFN, interferon; STAT, signal transducer and activator of transcription; IRF, interferon regulatory factor; ISGF3, interferon-stimulated gene factor 3; GAF, γ-activated factor; ISRE, interferon-stimulated response element; GAS, γ-activated sequence; ISG, interferon-stimulated gene.

Closer inspection of the publicly accessible dataset (111) featuring STAT1, STAT2, and IRF1 ChIP-seq experiments performed on chromatin extracted from K562 cells treated with IFNα or IFNγ for 30 min or 6 h (www.encodeproject.org), could recognize the binding to typical ISRE or GAS-containing genes of known and novel origin (Table 1; Figure 4). In addition, it revealed the existence of a large group of IFN-I and IFN-II activated genes that use ISRE and GAS composite sites (see Table 1). These observations were in line with our previous generated ChIP-seq dataset that examined binding of STAT1, STAT2, and IRF9 in IFNα-treated and untreated 2fTGH cells (Figure 4; Bluyssen et al., manuscript in preparation). The ISRE-only genes, *ISG15, MX1, OAS3*, and *IFIT3* clearly only bind ISGF3 in response to IFN-I (as represented by co-binding of STAT1, STAT2, and IRF9) as well as IRF1 after IFN-I and IFN-II stimulation (Figure 4), whereas the GAS-only genes (i.e., *IRF1, SOCS3*, and *ICAM1*) strictly bind GAF and/or pSTAT1–STAT2 under these conditions (Figure 4). By contrast, the ISRE and GAS composite-containing genes, exemplified by *IFI35, DTX3L, TRIM69, AIM2, BST2, SOCS1, STAT1, STAT2*, and *IRF9*, display a binding pattern of STAT1, STAT2, IRF9, and IRF1 that points to a mechanism of co-binding of ISGF3, IRF1, or GAF complexes in response to IFN-I or IFN-II (Figure 4). The presence of these different ISG sub-groups is in agreement with previous data (107, 112). Hassan et al. further highlighted the importance of STAT1 and IRF1 cooperation by detailed studies of ISG-rich chromosomal segments (containing ~10% of all known ISGs) in HeLa cells. Under these conditions, most ISG-rich loci responded to IFNγ, with only ~20% of ISGs being unresponsive. IRF1 binding sites were detected twice as often as STAT1 sites, with isolated IRF1 binding as a consequence. On the other hand, most STAT1 binding occurred at or near to IRF1 sites (dual binding), which was closely linked to ISG responsiveness (75).

Presently it is not clear how these ISRE and GAS composite-containing genes are represented within the full spectrum of IFN-I and IFN-II induced ISGs and how they contribute to overall IFN-mediated antiviral activities.

At the same time, these genome-wide binding approaches concluded that STAT1, STAT2, and IRF9 bind not only at promoter proximal but also distal ISRE and/or GAS sites suggesting a role of distant enhancers in remote gene regulation (83). Indeed, it was shown before that IFNγ induces long-range interactions between STAT1-bound enhancers and target promoters (75, 113–115). Moreover, several studies reported the functionality of typical enhancers, involving long-range interactions and IFN-induced recruitment of STAT1 to distal regulatory regions. For example, Li et al. showed for the *IFITM1, 2*, and *3* gene cluster that STAT1 binding (and possibly that of STAT2) to the E2–3 enhancer, which was located >35 kb away from the *IFITM* gene cluster, was indispensable for its IFN-responsive enhancer activity (116). It is quite interesting that the promoters of *IFITM* genes already contain the binding sites for STAT1 or STAT2 while there still exists an enhancer mediating IFN/STAT signaling. A reasonable explanation might be that the distal enhancer could fine-tune the expression of these important genes and thus add robust and plastic response to virus invasion and IFN stimulation. Likewise, Yuasa and Hijikata (117) identified a novel distal regulatory element (5.5URR) positioned 5.5-kb upstream of the mouse STAT1 gene (117). The 5.5URR is evolutionary highly conserved in its upstream localization of the STAT1 gene and presence of a combined ISRE and a GAS site (118). By association of STAT1 complexes, namely ISGF3 and GAF, this ISRE and GAS composite was shown to physically associate with the STAT1 core promoter and proposed to mediate the autoregulation of the STAT1 gene and its prolonged expression in response to IFN-I and IFN-II (117). Examination of the above described ChIP-seq datasets (K562 and 2fTGH), identified the presence of the 5.5URR enhancer also in the human STAT1 gene with apparent co-binding of ISGF3, GAF, and/or IRF1 in response to both types of IFN (Table 1; Figure 4).

ChIP-chip and ChIP-seq studies also showed that IRF1 binds many distal ISRE-containing enhancers (75, 83, 119–121). For example, IRF1 binds to remote enhancers of the *CIITA* locus that form a 3D interconnected loop with the promoter (115). Similarly, functionality of putative distal enhancers at the *SOCS1* locus were confirmed, combined with a co-binding role of IRF1 and STAT1 (75). In particular, the distal enhancer at −55 kb from the transcriptional start site of the human SOCS1 gene was indispensable for its IFN-responsive enhancer activity. According to the above described ChIP-seq datasets (K562 and 2fTGH), a comparable binding pattern of STAT1 and IRF1 could be observed to this distal ISRE and GAS-containing enhancer of *SOCS1* (Table 1; Figure 4).

The function of the majority of distal ISGF3, GAF, and IRF1 binding sites remains largely unknown. Nevertheless, it predicts the presence of a common regulatory mechanism of ISG transcriptional regulation.

### IFN Independent

IFN independent, basal expression of some ISGs has recently been linked to the nuclear localization of U-ISGF3 and U-GAF (56, 57). Accordingly, Wang et al. extracted genome-wide STAT1 ChIP-seq data (GSE31477) from the ENCODE ChIP-seq Experiment Matrix database and Gene Expression Omnibus (111) and observed that even in the absence of IFN stimulation STAT1 displayed specific binding to promoter regions of an extensive group of ISGs (186 of 350 ISGs analyzed), including *IRF1, IRF9, STAT1*, and *ISG15* (55). As becomes clear from Figure 4, after further examination of this dataset (GSE31477) (and to a lesser extent from our 2fTGH dataset), a selection of ISRE-only (ISG15 and MX1), GAS-only (*IRF1* and *ICAM1*), and ISRE GAS composite genes (*IFI35, TRIM69, AIM2, STAT1, STAT2*, and *IRF9*) display binding of STAT1 under basal conditions. This is in agreement with a predicted role of U-ISGF3 or U-STAT1 in IFN-independent ISG expression (55, 75). At the same time, these observations coincide with the fact that this is not a general phenomenon for all ISGs, but apparently concerns a selected group (55).

Interferon regulatory factor 1 is also expressed at low levels in many unstimulated cell types, including HeLa cells (115), and it collaborates with U-STAT1 in maintaining low basal expression of for example *LMP2* (56). Likewise, constitutive IRF1 binding facilitates constitutive *PSMB9* and *TAP2* expression (56, 122). Using ChIP-ChIP, Hassan et al. detected a number of U-STAT1 and IRF1 binding sites in untreated cells, accounting for 2.2 and 14.3% of induced sites, respectively (75). In addition, the basal expression of all of these genes was confirmed by microarray and/or RT-PCR (75). These data accord with another ChIP-chip analysis of STAT1 binding in HeLa cells treated for 30 min with IFNγ, observing 6.5% of IFNγ-induced STAT1 sites being occupied in unstimulated cells (123). In addition, basal TF binding loci were present in paralogous gene clusters predicting a link between gene duplication and high affinity binding site conservation (e.g., *PSMB8* and *PSMB9, GBP2* and *GBP3*, and *IFIT1, IFIT2*, and *IFIT3*). Moreover, the majority of the IRF1 basally occupied sites possessed ISRE motifs (75).

In a recent ChIP-chip study by Testoni et al., using anti-STAT2 and anti-phosphoSTAT2 antibodies, U-STAT2 was shown to be present in the nucleus of untreated cells and already bound to 62% of its target promoters, including many “classical” ISGs (87). This implies that under basal conditions nuclear U-STAT2/IRF9 binds genome-wide to ISG ISREs, including those encoding ISGF3 components, and keeping their expression at a low level.

Thus, increasing evidence supports the notion that basal genome-wide binding of U-ISGF3, U-STAT2/IRF9, U-GAF, and IRF1 is physiologically relevant.

## ISRE and Gas-Dependent Regulation of the STAT1, STAT2, IRF9, and IRF1 Genes: A Model of Positive Feedback Regulation

### IRF1

As mentioned above, transcriptional regulation of the *IRF1* gene in response to IFN-I and IFN-II depends on a single GAS element. According to the above-described ChIP-seq datasets (K562 and 2fTGH), clear binding of STAT1 and STAT2, but no IRF9 or IRF1, was observed at the same position in the proximal *IRF1* promoter (Figure 4; left peak) in a time-dependent manner. This corresponds with the presence of a single GAS element and no ISRE in the *IRF1* proximal promoter (Table 1) and with previous studies (62, 67), in which a heterodimer of STAT1 and STAT2 (without IRF9) was shown to bind to this site and regulate expression of the *IRF1* gene (62) in response to IFN-I. Moreover, binding of STAT1 (but not IRF1) to this proximal GAS in the *IRF1* promoter in response to IFN-II (Figure 4; K562 cells only), is in line with the functional role of a STAT1 homodimer-mediated transcription (95). Closer examination, identified a similar binding pattern of STAT1, STAT2, IRF9, and IRF1 in response to IFN-I and IFN-II in the two different cell lines on more distal sites in the *IRF1* promoter (Figure 4: right peaks; Table 1: first and second distal), which likewise corresponded with the presence of putative GAS sites in the sequences under the peak (Table 1). The functionality of these sites in relation to IRF1 expression is currently not known. Basal binding of U-STAT1 to the *IRF1* proximal promoter, as observed in the GSE31477 dataset (Figure 4), correlates with IFN-independent expression of IRF1 (Figure 3) (75).

### STAT1, STAT2, and IRF9

In many cell types, expression of STAT1, STAT2, and IRF9 is increased in response to IFN-I and IFN-II, being detectable even several days after treatment (10–12, 107). This IFN-dependent expression relies on the presence of ISRE and GAS-containing regulatory sequences.

In relation to the *STAT1* gene, several elements have shown to be involved in its transcriptional regulation in response to IFN-I and IFN-II: a GAS and ISRE site within the *STAT1* proximal promoter (87, 117, 124); the upstream region of exon 1 containing an ISRE site (125); an IRF-binding element (IRF-E)/GAS/IRF-E (IGI) motif at the intron 1/exon 2 boundary region (97): the recently identified distal ISRE and GAS-containing enhancer (5.5URR) (117).

The regulatory regions of the *STAT2* and *IRF9* genes were studied less extensive. According to Yan et al., the proximal promoter of *STAT2* contains a functional ISRE, but no GAS (98). However, sequence alignment of the mouse and human genome sequence revealed conserved GAS-like and ISRE elements in the promoter that corresponded with IFN-I and IFN-II responsiveness (87, 117). Likewise, *IRF9* harbors ISRE and GAS elements in its promoter, which are targets of ISGF3 and GAF in response to IFN-I or IFN-II (87, 117).

By using the K562 and 2fTGH ChIP-seq datasets, comparative ISGF3, GAF, and IRF1 binding patterns could also be observed for the *STAT1, STAT2*, and *IRF9* genes (Figure 4). For all of these genes STAT1 and STAT2 binding as well as IRF9, was observed in the promoter (Figure 4; Table 1; STAT1: distal; STAT2 and IRF9: proximal) in a time-dependent manner (even after 24 h) in 2fTGH and K562 IFN-I treated cells. This corresponds with the presence of an ISRE and a GAS element in close proximity in their promoters (Table 1). Under these conditions, IRF1 binding could be detected to *STAT1* and *STAT2* genes, and weakly to *IRF9*, which further substantiates the observation that *STAT1, STAT2*, and *IRF9* are IRF1-targets (48). Binding of STAT1 and IRF1 to these promoter sites in the *STAT1, STAT2*, and *IRF9* genes in response to IFN-II (Figure 4; K562 cells only), is in agreement with the presence of a functional GAS and ISRE element in mediating their IFN-II-induced transcription (97, 98, 117). Recently, a novel distal regulatory element was described positioned 5.5-kb upstream of the mouse Stat1 gene (117) with a similar ISRE and GAS composite structure as we observe here in the human gene (Figure 4: right peak; Table 1: distal). By association of STAT1 complexes, namely ISGF3 and GAF, this ISRE and GAS composite was proposed to mediate the autoregulation of *STAT1* gene and its prolonged expression in response to IFN-I and IFN-II (117). The observations in Figure 4 are in agreement with this idea for STAT1 and confirm the presence of a similar ISRE and GAS organization for the human *STAT1, STAT2*, and *IRF9* genes.

Closer examination identified co-binding of STAT1, STAT2, IRF9, and/or IRF1 in response to IFN-I and IFN-II in the different cell lines on a proximal site in the *STAT1* promoter (Figure 4: left peak; Table 1: proximal), which likewise corresponded with the presence of putative ISRE site in the sequence under the peak, but no GAS could be recognized. Similarly, for the *STAT2* gene a second STAT1, STAT2, and IRF9 binding peak could be observed in 2fTGH, but not (or very weak) in K562 (Figure 4: left peak), corresponding to a putative ISRE site (Table 1: intronic). The functionality of these sites in relation to STAT1 and STAT2 expression are currently not known. Basal binding of U-STAT1 to the *STAT1, STAT2*, and *IRF9* promoters, as observed in the GM12878 cell line (Figure 4), correlates with IFN-independent expression of these genes (Figure 2) (55).

Together, the genomic organization and the binding pattern of ISGF3, GAF, and IRF1, predict the involvement of co-binding and provide an explanation for the positive feedback regulation in IFN-I and IFN-II activated expression of the *STAT1, STAT2* and *IRF9* and *IRF1* genes.

## Positive Feedback Regulation of ISGF3 and GAF Components and IRF1 in Progression and Maintenance of IFN-I and IFN-II Responses

**Figure 5:**
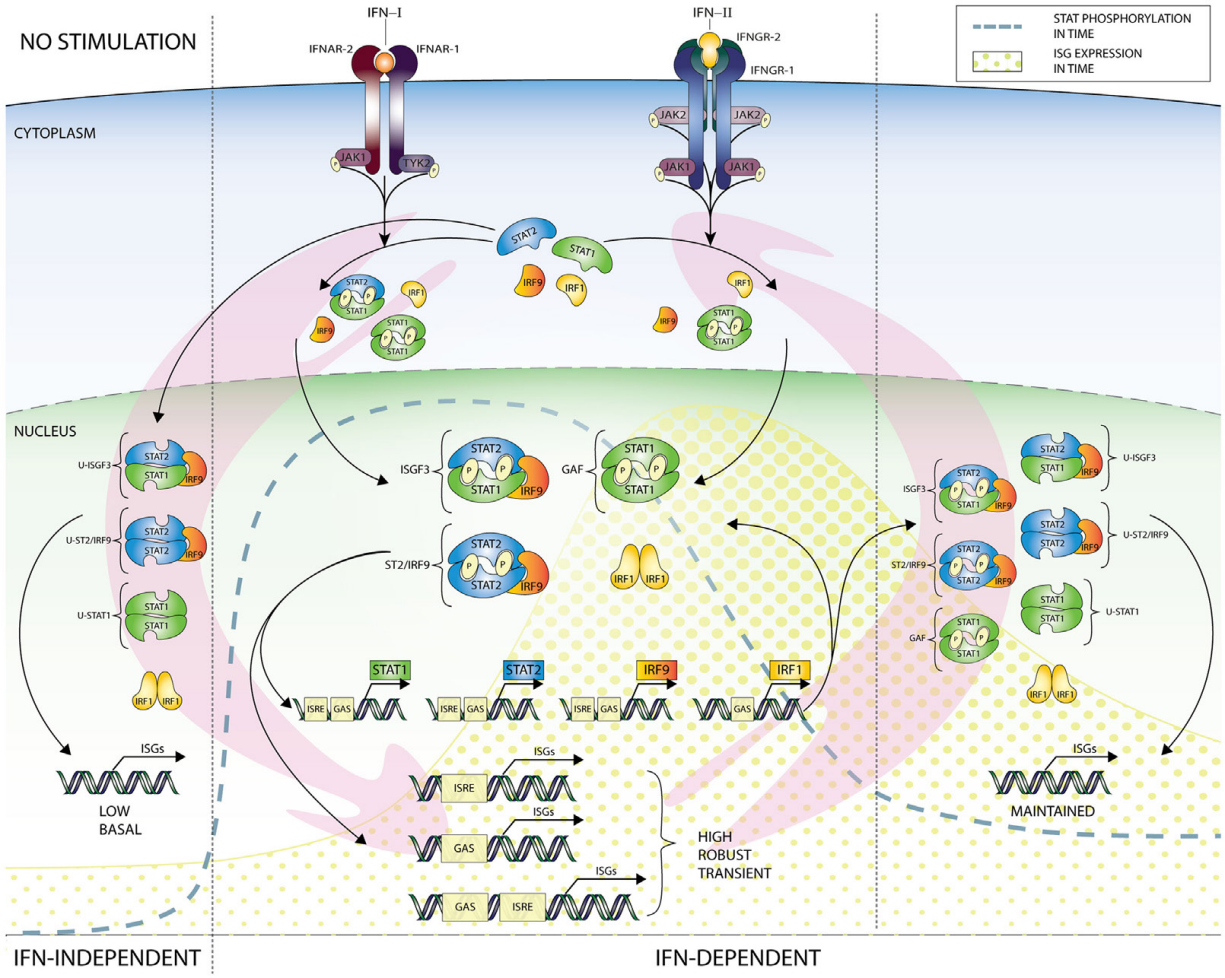
Cooperation of unphosphorylated and phosphorylated ISGF3 and GAF components and IRF1 in the regulation of basal and IFN-induced transcription. In unstimulated cells, most of the known ISGs are expressed at low but detectable levels. Among these genes, there are examples of components (STATs and IRFs) of transcription factors, which in the absence of stimulation can combine in unphosphorylated complexes (U-ISGF3, U-STAT1, U-STAT2/IRF9, and IRF1) and drive the constitutive expression of ISGs. After cell stimulation with IFN type I or II, receptors dimerize and enable receptor-bound kinases (JAK1 and TYK2 for IFN-I or JAK1 and JAK2 for IFN-II) transphosphorylation and STAT protein recruitment. Next step is phosphorylation of STATs, which can now dimerize and with or without a partner (IRF9) create complexes—ISGF3, ST2/IRF9, and GAF, then translocate to the nucleus and start a high and robust but transient expression (yellow dotted graph) of GAS and/or ISRE-containing ISGs. This leads to, *inter alia*, rapid accumulation of newly synthesized STAT1, STAT2, IRF9, and IRF1 proteins in the cytoplasm, which can create new transcription factors in unphosphorylated form (U-ISGF3, U-STAT1, and U-STAT2/IRF9), and while level of phospho-proteins is dropping (dotted line) support or take over the role of phosphorylated complexes in sustaining the expression of ISGs. Important role in the circuit plays IRF1, which can occupy ISRE-containing genes and co-binds with other factors to modulate IFN-induced response, as well as basal ISG expression. As shown in the graph, responses to both types of IFN rely on common elements and events of the JAK–STAT signaling pathway and result in partially shared outcome. Thus, IFN-I and IFN-II induce a common as well as a unique set of genes. These facts clearly point to functional and biological overlap between IFN-I and IFN-II action and additionally to existence of mechanisms allowing cells to manage and adjust both responses. Abbreviations: IFN, interferon; STAT, signal transducer and activator of transcription; IRF, interferon regulatory factor; JAK, Janus kinase; TYK, tyrosine kinase; ISGF3, interferon-stimulated gene factor 3; GAF, γ-activated factor; ISRE, interferon-stimulated response element; GAS, γ-activated sequence; ISG, interferon-stimulated gene; P, phosphate; U, unphosphorylated form.

Based on the above, a picture emerges of a more complex and multifaceted mechanism of IFN-I and IFN-II-activated ISG regulation in which STAT1 and STAT2-containing ISGF3 and GAF-like complexes and IRF1 are recruited to individual or combined ISRE and GAS sites. This mechanism closely depends on positive feedback regulation of the STAT1, STAT2, IRF9, and IRF1 genes and facilitates long-term responses to IFN-I and IFN-II (Figure 5, central panel). In detail, IFN-I phosphorylates STAT1 and STAT2 and triggers the formation of STAT1 homodimers and STAT1–STAT2 heterodimers as well as ISGF3 that bind to ISRE and GAS sites in ISG promoters. IFN-II only phosphorylates STAT1 that forms homodimers to bind GAS sites. IFN-I and IFN-II also increase IRF1 expression through binding of STAT1 homodimers and STAT1–STAT2 heterodimers to a single GAS element in its promoter. On the one hand, positive feedback regulation of the *STAT1, STAT2, IRF9*, and *IRF1* genes is achieved through binding of these complexes to combined ISRE and GAS-containing regulatory sequences (Figure 5, central panel). In IFN-I signaling ISGF3 and IRF1 bind the ISRE and STAT1 homodimers and possibly STAT1–STAT2 heterodimers the GAS. In IFN-II action, IRF1 binds the ISRE and STAT1 homodimers the GAS. This leads to increased STAT1, STAT2, IRF9, and IRF1 protein expression, further enhancing complex formation in an IFN-I and -II dependent manner (Figure 5, central panel). On the other hand, these complexes participate in the increased expression of other ISRE and/or GAS-containing ISGs (Figure 5, central panel).

Strong evidence exists that the IFN-dependent positive feedback regulation of STAT1, STAT2, IRF9, and IRF1 depends on phosphorylated ISGF3 and GAF components in the early response phase. For example, STAT1 KO U3A cells rescued with tyrosine 701 mutated STAT1 (Y to F) (53) or STAT1 KO mice overexpressing this STAT1 Y to F mutant (19), lack this positive feedback system. Likewise, STAT1 KO U3C cells, which also have low levels of STAT2, lack this positive feedback system and are unresponsive to IFN treatment and unable to protect against viral infection (30). However, STAT1 KO U3C cells overexpressing STAT2 regain the IFN-mediated positive feedback regulation of IRF9, IFN-I responsiveness and antiviral activity (30). Contrary, macrophages from STAT1 KO mice (32) as well as Huh-7.5 STAT1 mutant cells (12), which express normal STAT2 amounts, still exhibit this feedback system. This implies that a level of redundancy exists between the individual ISGF3 and GAF components, but also that a certain threshold level must be reached, to ensure positive feedback of their expression and progressive responses to IFNs.

The exact timely steps that take place during IFN-activated feedback regulation and the control of ISG transcription and long-term cellular responsiveness to IFN-I and IFN-II, is currently not clear.

One possible mechanism describes the involvement of an ISGF3-like complex composed of unphosphorylated STAT1 and 2 with IRF9 (named U-ISGF3) or U-GAF in long-term treated cells where increased levels of unphosporylated ISGF3 components highly dominate their phosphorylated counterparts (10–12, 107). As such, U-ISGF3 is proposed to switch with ISGF3 in time to drive prolonged expression of ISGs, including STAT1, STAT2, and IRF9, in response to IFN-I. Similarly, a switch of U-STAT1 with GAF can be envisioned, under these conditions (Figure 5, right panel). Interestingly, macrophages from STAT1 KO mice (32) as well as Huh-7.5 STAT1 mutant cells (12) exhibit increased expression of U-STAT2 and IRF9 upon long-term IFN-I treatment. Therefore, a possible role exists for U-STAT2/IRF9 in the durable expression of typical IFN-I activated ISGs (31). The expression of ISGF3 components is also highly increased in long-term IFN-II treated cells (11), providing the possibility for the formation of U-ISGF3 and U-GAF as well. Therefore, a regulatory role for U-ISGF3 and U-GAF in long-term IFN-II-treated cells might also exist (Figure 5, right panel), however, currently no experimental evidence exists for this scenario.

A second mechanism predicts the involvement of phosphorylated STAT1 and STAT2 together with IRF9 (as ISGF3 or STAT2/IRF9) and phosphorylated STAT1 (as GAF) in positive feedback regulation and prolonged ISG expression in response to IFN-I and IFN-II (Figure 5, right panel). This is in agreement with the observation, that in many cell types levels of phosphorylated STAT1 and STAT2 drop from high to low upon long-term IFN-I and IFN-II treatment, being still detectable and maintained for several days (11, 112). At the same time, it correlates with the sustained expression of a large group of ISGs, including STAT1, STAT2, and IRF9, which follow the timely phosphorylation pattern of STAT1 and STAT2 (Figure 5, central panel). Likewise, the prolonged IFN-I activated phosphorylation of STAT2 in the absence of STAT1, strongly correlates with sustained ISG expression (12, 30, 32). Also, a novel role of ISGF3^II^, which consists of pSTAT1 together with U-STAT2 and IRF9, was proposed to be involved in long-term IFN-II signaling (11). Moreover, Yuasa et al., recently provided more insight into the autoregulatory mechanism of IFN-dependent STAT1 gene expression. They established that a novel distal regulatory element, 5.5URR, can mediate activation of the STAT1 gene, in an IFN-dependent manner. Indeed, by association of IFN-dependent STAT1 complexes, this enhancer was shown to physically associate with the *STAT1* core promoter and regulate STAT1 gene expression in response to IFN-I and IFN-II (117). The ChIP-seq data investigation of K562 and 2fTGH cells (see above) are in agreement with this idea for STAT1 and provide a similar ISRE and GAS-dependent mechanism for the positive feedback regulation of STAT2 and IRF9 (Figure 4).

Third, as a STAT1-specific target gene *IRF1* is highly induced in the early stages of IFN-I and IFN-II responses, while its levels are sustained upon long-term treatment following STAT1 phosphorylation (38). As such, IRF1 facilitates secondary responses to IFN-I and IFN-II in many cells. Among the target genes of IRF1 are *STAT1, STAT2*, and *IRF9* and a diversity of other ISRE-containing ISGs, agreeing with a functional overlap with ISGF3 (48, 61). It also suggests the involvement of IRF1 in the feedback regulation of ISGF3 and GAF components as well as the sustained expression of typical antiviral ISGs (Figure 5, right panel).

Finally, a novel function for U-STAT1, U-STAT2, IRF9, and IRF1 is appearing in untreated cells as U-ISGF3, U-STAT2/IRF9, U-GAF, or IRF1 DNA-binding complexes to keep ISG expression at a low basal level (55, 75) (Figure 5, left panel). Since many ISGs represent key components of antiviral pathways, their basal expression is essential for the robust activation of these IFN-activated signaling pathways. Moreover, basal binding of these complexes could possibly mark the genome for rapid ISGF3, STAT2/IRF9, GAF, and/or IRF1 recruitment upon IFN-I and IFN-II treatment and prepare cells for a robust and effective response against invading pathogens.

## Conclusion

According to the general paradigm, phosphorylation of STAT1 and STAT2 in response to IFN-I and/or IFN-II displays a robust and transient character. This is followed by a similar ISG expression pattern that decreases over time. However, numerous studies have shown that IFN signaling is much more complex and revealed that ISG expression patterns are globally sustained in response to both types of IFN (10–12). This sustained response relies on prolonged expression of the ISGF3 and GAF components STAT1, STAT2, and IRF9 and IRF1 as part of a positive feedback loop (Figure 5).

Together, this predicts the existence of a multifaceted intracellular amplifier circuit that depends on unphosphorylated and phosphorylated ISGF3 and GAF complexes and IRF1. In a combinatorial and timely fashion, these complexes mediate prolonged ISG expression and control cellular responsiveness to IFN-I and IFN-II (Figure 5). This proposed intracellular amplifier circuit also provides a molecular explanation for the existing overlap between IFN-I and IFN-II activated ISG expression.

The exact timely steps that take place during IFN-activated feedback regulation and the control of ISG transcription and long-term cellular responsiveness to IFN-I and IFN-II, are still not clear. The same holds true for the distinct functions of unphosporylated ISGF3 and GAF complexes in relation to their phosphorylated counterparts, especially in the transition from early to sustained IFN actions. For this, more detailed time-dependent genome-wide expression and chromatin interaction studies are required in wild type as compared to knockout back grounds of ISGF3 and GAF components and IRF1. This will allow discrimination between IFN-independent and IFN-dependent conditions, and between U-STAT and pSTAT regulatory functions and binding characteristics on promoter proximal and distal ISRE and GAS-containing sites. Moreover, it will provide insight in the role of the different components in the complex IFN-independent and IFN-dependent transcriptional control mechanisms involved in early and sustained ISG transcription.

## Author Contributions

AM was involved in concept development and writing and editing the manuscript, analyzed published ChIP-seq data, and designed the figures and table. KB generated novel ChIP-seq data and provided critical feedback on the manuscript. JW participated in development of the concept and critically evaluated and edited the manuscript. HB developed the concept and was involved in writing and editing the manuscript and coordinated input from all co-authors.

## Conflict of Interest Statement

The authors declare that the research was conducted in the absence of any commercial or financial relationships that could be construed as a potential conflict of interest.
